# Ectopic Overexpression of PPARγ2 in the Heart Determines Differences in Hypertrophic Cardiomyopathy After Treatment With Different Thiazolidinediones in a Mouse Model of Diabetes

**DOI:** 10.3389/fphar.2021.683156

**Published:** 2021-07-07

**Authors:** Xuemei Cao, Min Mao, Junlin Diao, Yi Hou, Hong Su, Yongjun Gan, Jibin Li, Xiaoyong Tong, Chaodong Wu, Zhong Zuo, Xiaoqiu Xiao

**Affiliations:** ^1^ Department of Endocrinology, The First Affiliated Hospital of Chongqing Medical University, Chongqing, China; ^2^ The Chongqing Key Laboratory of Translational Medicine in Major Metabolic Diseases, The First Affiliated Hospital of Chongqing Medical University, Chongqing, China; ^3^ Department of Cardiology, The First Affiliated Hospital of Chongqing Medical University, Chongqing, China; ^4^ Experimental Teaching & Management Center, Chongqing Medical University, Chongqing, China; ^5^ Department of Nutrition and Food Hygiene, School of Public Health and Management, Chongqing Medical University, Chongqing, China; ^6^ School of Pharmaceutical Sciences, Chongqing University, Chongqing, China; ^7^ Department of Nutrition and Food Science, Texas A&M University, College Station, TX, United States

**Keywords:** cardiac hypertrophy, diabetic cardiomyopathy, insulin sensitizers, PPARγ, rosiglitazone

## Abstract

The clinical controversy of rosiglitazone as a hypoglycemic agent is potentially associated with heart failure, mainly due to its potent activation of peroxisome proliferator-activated receptor γ (PPARγ). PPARγ partial agonists showed superior pharmacological profiles to rosiglitazone. This study compared differences in cardiac morphology and function of the PPARγ partial agonist CMHX008 with rosiglitazone. High-fat diet (HFD) induced obese mice, ob/ob mice and cardiomyocytes overexpressing PPARγ2 were treated with CMHX008 or rosiglitazone. Heart function, myocardial morphology, and hypertrophy-related gene expression were examined. Clinical information from patients with type 2 diabetes mellitus (T2DM) who had taken rosiglitazone and undergone Doppler echocardiography was collected. HFD and ob/ob mice significantly developed cardiac contractile dysfunction, with upregulated PPARγ2 protein levels in heart tissues. Cardiomyocytes of HFD and ob/ob mice were disorderly arranged, the cell areas expanded, and collagen accumulated. *In vitro* cardiomyocytes overexpressing PPARγ2 displayed obvious structural abnormalities and high mRNA levels of ANP and BNP, critical cardiac hypertrophy-related genes. HFD-fed mice treated with rosiglitazone or CMHX008 had significantly improved cardiac function, but rosiglitazone induced higher expression of ANP and βMHC and hypertrophic cardiomyopathy, while CMHX008 did not. Patients with T2DM taking rosiglitazone exhibited increased thickness of the posterior wall and the ventricular septum, suggesting cardiac hypertrophy. Our findings show that diabetic cardiomyopathy was associated with ectopic overexpression of PPARγ2. The full agonist rosiglitazone prevents cardiac dysfunction at the expense of compensatory hypertrophy, while the partial agonist CMHX008 shared a comparable protective effect without altering the structure of cardiomyocytes.

## Introduction

Diabetes as a chronic, metabolic disease has recently attracted widespread attention. As WHO showed, type 2 diabetes mellitus (T2DM) is believed to account for approximately 90% of all cases of diabetes, which is caused by insulin resistance or insufficient insulin. Obesity-induced insulin resistance is associated with T2DM (Saltiel, 2006; Malone and Hansen, 2019). Thiazolidinediones (TZDs) as insulin sensitizers are important hypoglycemic agents for T2DM (Eldor et al., 2013; Nanjan et al., 2018). TZDs, including rosiglitazone and pioglitazone, are synthetic full agonists of peroxisome proliferators activated receptor γ (PPARγ), and can activate PPARγ to better combine with insulin receptors for maintaining glucose levels and increasing adiponectin expression in patients with diabetes (Yki-Järvinen, 2004; Rizos et al., 2016). However, activating PPARγ by full agonists is accompanied by some side effects, most remarkably, congestive heart failure caused by rosiglitazone (Home et al., 2009; MacDonald et al., 2011). Although careful safety evaluations demonstrated that rosiglitazone was with no increased risk of heart attack compared to the standard T2DM medicines metformin and sulfonylurea, great concerns remained as to the potential side effects of classical TZDs (Consoli and Formoso, 2013; Hiatt et al., 2013; Goltsman et al., 2016). In order to reduce these side effects, such as heart problems, bone loss, weight gain, and fluid retention (Komajda et al., 2010; Lecka-Czernik, 2010; Ahmadian et al., 2013; Stechschulte et al., 2016; Lebovitz, 2019), several strategies have been examined and accumulating evidence has suggested that novel PPARγ agonists that do not fully stimulate PPARγ can also play an important role in enhancing insulin sensitivity (Ryan et al., 2011; Kolli et al., 2014; Yasmin et al., 2017). Studies have also demonstrated that partial agonists retain insulin sensitization with lower damage to metabolic organs (Wang et al., 2014; Chigurupati et al., 2015). A novel PPARγ partial agonist, SPPARγM5, increased insulin sensitization and reduced the cardiovascular side effects (Chang et al., 2008). Another study showed that the CKD-501 compound has better safety in the cardiotoxicity of db/db mice than full PPARγ agonists (Yang et al., 2013). Thus, it is postulated that PPARγ partial agonists can be developed into a more ideal antidiabetic agent. In this study, the structure of TZDs was modified to synthesize a novel compound, namely, CMHX008, which has been identified to have a lower PPARγ agonist activity, promote preadipocyte differentiation, and improve insulin sensitivity, with minor influence on weight gain and bone loss compared with rosiglitazone (Ming et al., 2014; Hou et al., 2018). These findings revealed CMHX008 as a new PPARγ partial agonist; thus, it was expected to have a similar efficacy in increasing glucose uptake and potential effects on the risk of cardiovascular events as rosiglitazone. Therefore, we will further explore the underlying mechanism of cardiac hypertrophy after treatment with CMHX008 and rosiglitazone.

PPARγ is a ligand-inducible nuclear hormone receptor superfamily that controls gene expression linked to a variety of physiological processes (Janani and Ranjitha Kumari, 2015; Hall et al., 2020). PPARγ exists as two isoforms, PPARγ1 and PPARγ2, encoded by two isoforms of genes PPARG1 and PPARG2 with alternative use of two different promoters (Semple et al., 2006). Compared with PPARγ1, PPARγ2 has an additional 30 AA in the N terminus sequence. PPARγ1 is widely expressed in several key metabolic organs, while PPARγ2 is relatively specific to adipose tissues and is a master regulator for the development of adipose cells under physiological conditions. The expression patterns and biological functions of PPARγ on cardiomyocytes were not fully understood. Previous study demonstrated that after high expression of PPARγ1 specifically in cardiomyocyte, fatty acid and glycogen were accumulated in the heart tissue, and mice showed hypertrophic cardiomyopathy (Son et al., 2007). However, the mechanism underlying the effect of PPARγ2 on heart function remains to be elucidated. For this reason, we focused on examining whether PPARγ2, the regulator of fatty acid-related factors, was a contributor to diabetic cardiomyopathy.

## Materials and Methods

### Animals and Treatment

Six-week-old male C57BL/6 mice were purchased from the Experimental Animal Center of Chongqing Medical University. Mice were randomly divided into two groups, and each group was fed a control low-fat diet (LFD, with 10% of calories from fat, D12450B; Research Diet) or a high-fat diet (HFD, 60% of calories from fat, D12492; Research Diet) for 12 weeks in SPF conditions. Mice on HFD feeding did not reach the criteria for diet induced obesity (Enriori et al., 2007) were eliminated from the following studies. Beginning with 13-weeks on HFD, obese mice were randomly divided into five groups and switched to gavage daily with vehicle (HFD-Veh) or rosiglitazone (3 or 10 mg kg-1; HFD-R3 or HFD-R10) or CMHX008 (3 or 10 mg kg-1; HFD-C3, or HFD-C10), respectively. Ob/ob mice were generated by our lab. All mice were kept in temperature- (25°C) and humidity (60%)-controlled rooms on a 12/12-h light/dark cycle. The mice were weighed weekly to monitor their body weight.

### Color Doppler Ultrasonography Assay

The mice were subjected to abdominal hair removal treatment using a depilatory cream, anesthetized with isoflurane, and a ventilator (Harvard, United States) was used to maintain a stable heart rate. Color Doppler ultrasonography (Vivid 7 Dimension) was used to obtain the data from two-dimensional ultrasound. M-type ultrasonographic parameters are as follows: 1. left ventricular end-diastolic posterior wall thickness (LVPWd) and end-systolic posterior wall thickness (LVPWs); 2. left ventricular end-diastolic septal thickness (IVSd) and end-systolic septal thickness (IVSs); 3. left ventricular end-diastolic diameter (LVIDd) and end-systolic diameter (LVIDs) × 100%; 4. left ventricular fractional shortening (FS)= (LVIDd - LVIDs)/LVIDd × 100%; 5. ejection fraction (EF) = SV/LVEDV × 100%; and 6. mitral valve flow rate = E/A.

### Automatic Biochemistry Analyzer Assay

After administration of ether anesthesia, we obtained blood samples from mice from the eyes and kept it in 1.5 ml tubes coated with heparin on ice. The blood samples were centrifuged, and serum was kept for detection. Serum triglyceride (TG), total cholesterol (TC), high-density lipoprotein (HDL), low-density lipoprotein (LDL), and aspartate aminotransferase (AST) and alanine aminotransferase (ALT) were measured with Biochemical Analyzer (SIEMENS ADVIA 2,400 Chemisty Systerm, Germany).

### Wheat Germ Agglutinin Staining and Morphological Analysis

Heart tissue was fixed in 4% paraformaldehyde, and after 48 h, samples were paraffin embedded and sectioned (4 μm) for WGA and DAPI staining. Microscopic views of WGA-stained transverse sections of heart tissue were observed by microscopy, and ImageJ was used for analyse of pictures measuring the cell areas.

### Cell Culture and Treatment

Mouse cardiac myocytes (MCM) were purchased from the ScienCell Research Laboratories (M6200, Carlsbad, CA, United States) and cultured in Dulbecco’s modified Eagle’s medium (DMEM containing 4.5 g/L glucose; PAN Biotech, French) supplemented with 10% fetal bovine serum (FBS; PAN Biotech, Germany) and 1% Penicillin-Streptomycin Solution (Beyotime, China) maintained at 37°C in an atmosphere of 95% air and 5% CO2. Cells were digested with 1 ml of trypsin (HyClone, United States) at 37°C for 1–2 min. After digesting, cells were centrifuged for 3 min at a speed of 800 rpm, plated at 5 × 104 cells per well in 24-well plates, and incubated in DMEM containing 10% FBS for 24 h. Complexes of transfection reagent (Megran 1.0; Origene, United States) and DNA were made of 100 μl of DMEM, 1 μg of DNA, and 3 μl of transfection reagent, mixed, and incubated at room temperature for 10 min. Complexes (1 μg/ml) were added in 24 wells and incubated for 24 h to detect target mRNA and protein by RT-PCR and western blot. After successful transfection, the cells transfected with PCMV6-PPARγ2 (Origene, United States) were treated with 10 μmol/ml rosiglitazone or CMHX008 for 24 h.

### Quantitative Real-Time PCR

Heart tissues were stored in RNAstore Reagent (#Q5324, TIANGEN). Total RNA was extracted from heart tissues or cardiac myocytes using TRIzol reagent (Invitrogen, United States) according to the manufacturer’s instructions. cDNA was synthesized with the PrimeScriptTM RT Reagent Kit (TAKARA, Japan). RT-PCR was carried out using the SYBR Green premix Ex TaqⅡKit (Roche, Germany). Expression of PPARγ2, BNP, and β-MHC mRNA in heart tissues and BNP and ANP mRNA in cardiomyocytes was measured using RT-PCR. After the reaction system was completed, all gene levels were analyzed according to the Cq values. Primer sequences were as described in Table 1. The protocol of PCR was as follows: 95.0°C for 0.5 min, 95.0°C for 5 s, 60°C for 34 s, more than 39 cycles of 95.0°C for 5 s, with additional melt curve program included (Thornton and Basu, 2015).

**TABLE 1:** Primer information of quantitative real-time PCR experiments.

Gene name	Sequence 5′-3′ (forward)	Sequence 5′-3′ (reverse)
GAPDH	GAC​ATC​AAG​AAG​GTG​GTG​AAG​C	GAA​GGT​GGA​AGA​GTG​GGA​GTT
ANF	TAG​GAG​ACA​GTG​ACG​GAC​AA	GAAGAAGCCCAGGGTGAT
BNP	AGG​AAA​TGG​CCC​AGA​CAG​C	TTG​TGC​CAA​AGC​AGC​TTG​AG
β-MHC	CCG​AGT​CCC​AGG​TCA​ACA​A	CTT​CAC​GGG​CAC​CCT​TGG​A
PPARγ2	GACCACTCGCATTCCTTT	CCACAGACTCGGCACTCA

### Western Blotting

We stored heart tissues at −80°C. Proteins from tissues or cells were isolated and extracted using RIPA Buffer (89,900, Roche, Germany) with protease inhibitor (Roche, Germany). A BCA protein assay kit (P0010, Beyotime, China) was used to detect the protein concentration. Protein samples were separated in 8% denaturing sodium dodecyl sulfate polyacrylamide gels and transferred onto polyvinylidene fluoride membranes (Millipore, United States). Membranes were blocked with skimmed milk powder (Bio-Rad, United States) for 1.5 h and incubated at 4°C overnight with primary antibodies against PPARγ2 (1:1,000, ab45036, Abcam), DDK (1:2,000, TA50011, OriGene), and GAPDH (1:500, TA505454, ZSGB-Bio). Then, the membranes were washed in TBST 3 times, followed by incubation in secondary antibody (horseradish peroxidase-labeled lgG anti-goat/rabbit antibody; 1:2,000, OriGene, China) for 1 h at room temperature. After washing membranes in TBST 3 times, the western blotting detection kit (Millipore, United States) was used for exposure. Densitometric measurements of the bands were performed using FusionCapt Advance software (VILBER, French).

### Immunofluorescence Staining

The films were plated into 24-well plates and cultured with 5 × 104 cells per well in an incubator for 24 h. Subsequently, the cells were treated with plasmids and drugs for 48 h. Cells were fixed with 4% paraformaldehyde for 20 min and permeabilized with 0.1% Triton-X-100 at room temperature for 15 min. After blocking with 5% bovine serum albumin (GENView, United States) for 1 h, the cells were incubated with primary antibodies against α-SMA (1:100, Santa Cruz) 50 μl at 4°C overnight. Subsequently, cell nuclei were stained with DAPI for 1 min. We observed cells using fluorescence microscopy (Olympus AX70, Japan). The entire experimental process was carried out in the dark.

### Clinical Data Analysis

To test whether rosiglitazone treatment would cause cardiac hypertrophy, we collected the information of patients with T2DM from the Medical Reports Department of the First Affiliated Hospital of Chongqing Medical University in the recent three years (from 2017.01.01 to 2019.12.31). At first, we selected the patients with the examination of heart color doppler ultrasound and heart function. Then patients taking rosiglitazone as one of the combinational therapy were selected and the plasma biochemical index and color Doppler ultrasonography data were collected. Another group of age, sex, and disease course matched patients without taking rosiglitazone was used as a control to compare the differences in cardiac structure and function, such as ventricular wall thickness, ventricular septal thickness, ejection fraction, mitral valve flow rate, and etc.

### Statistical Analysis

We use SPSS18 software to analyze all data. Data values are presented as the mean ± SEM. Comparisons of two groups were made using the LSD test and one-way ANOVA. Statistical significance was expressed as *p < 0.05, **p < 0.01, and ***p < 0.001.

## Results

### Obesity Was Associated With Cardiac Dysfunction and Ectopically Upregulated PPARγ2 Expression in the Heart

**FIGURE 1:**
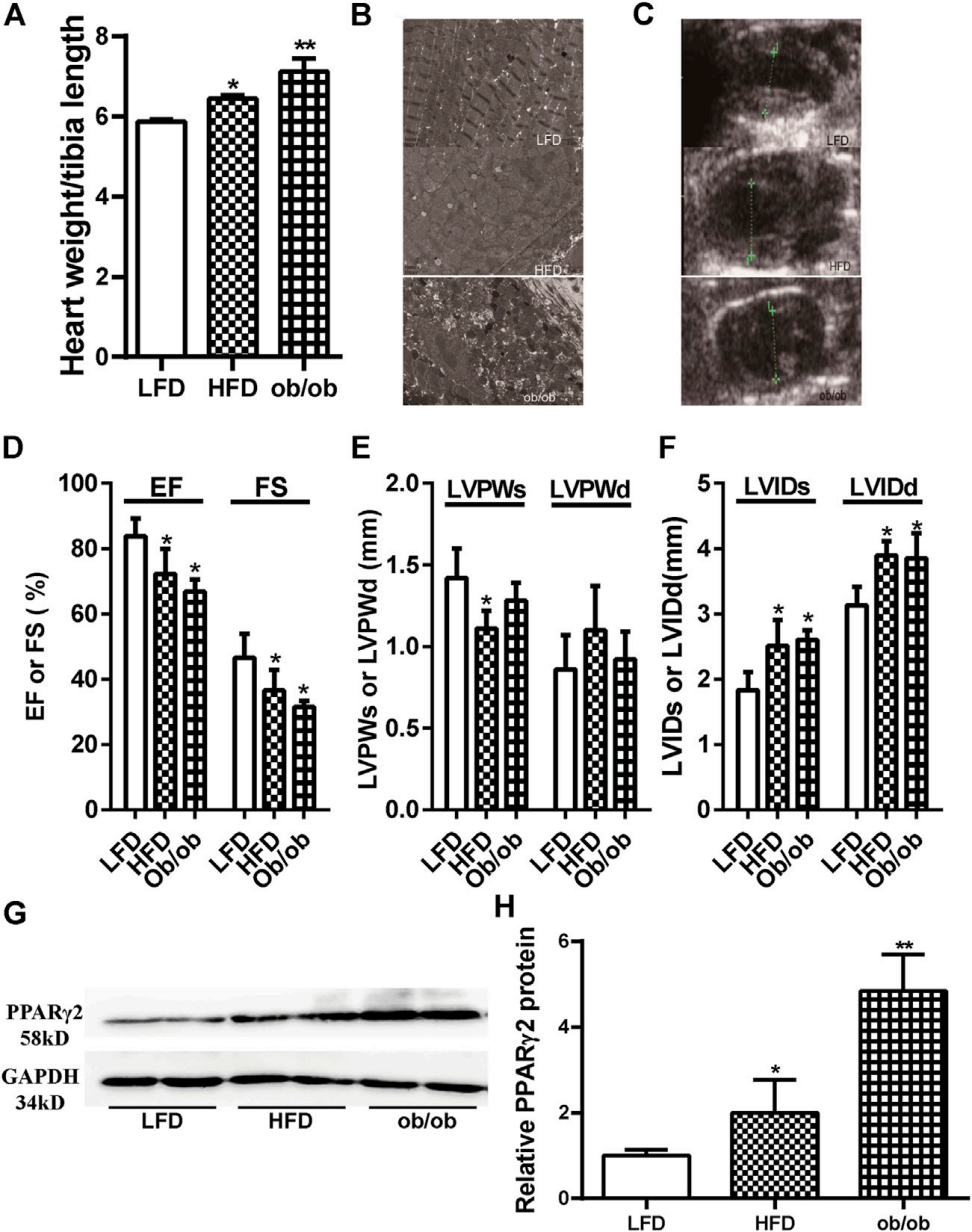
Obesity was associated with cardiac dysfunction and ectopically upregulated PPARγ2 expression in the heart. **(A)** The ratio of the heart weight to tibia length. **(B)** and **(C)** The representative 2D echocardiograph and M-mode image of lean and obese mice. **(D)** Quantification of ejection fraction (EF) and fractional shortening (FS). **(E)** Measurements of the left ventricle wall thickness in systole (LVPWs) and diastole (LVPWd). **(F)** The left ventricle internal diameters in systole (LVIDs) and diastole (LVIDd). **(G)** Representative PPARγ2 bands of Western blot in heart tissues. **(H)** Quantified protein levels of PPARγ2 in the heart of the LFD group or obese mice. Mean ± SEM, *n* = 5–8 mice per group, *p < 0.05, **p < 0.01 vs. LFD.

Obesity is a major risk factor for diabetes and cardiovascular diseases. The expression of PPARγ2 in heart tissues was evaluated in two models of obesity using C57BL/6 mice fed HFD for 12 weeks and genetic ob/ob mice. The ratio of the heart weight to tibia length was used as an indicator of cardiac size. The ratios in the HFD and ob/ob mice were significantly higher than those in the LFD mice (Figure 1A). Electron microscopy results showed that the mitochondria were swelled, cristae were ruptured, and endoplasmic reticulum was extended in the myocardium of HFD and ob/ob mice. In addition, muscle fibers were partially dissolved in the myocardium of ob/ob mice (Figure 1B). Compared with the LFD group, cardiac color Doppler ultrasonography showed that EF and FS decreased in the HFD and ob/ob groups, which indicated a decrease in contractility. Enlargement of the left ventricular diameter was also observed in the HFD group (Figures 1C–F). Although PPARγ2 regulates metabolic-related genes and is primarily expressed in adipocytes, we found that it was also expressed in myocardium. Compared with the LFD control, the HFD and ob/ob mice showed approximately a 1-fold and 4-fold increase at the PPARγ2 protein levels (Figures 1G,H). Thus, we believe that PPARγ2 is highly expressed in the heart of HFD mice and ob/ob mice.

### Overexpression of PPARγ2 in Cardiomyocytes is Sufficient to Lead to Hypertrophy

**FIGURE 2:**
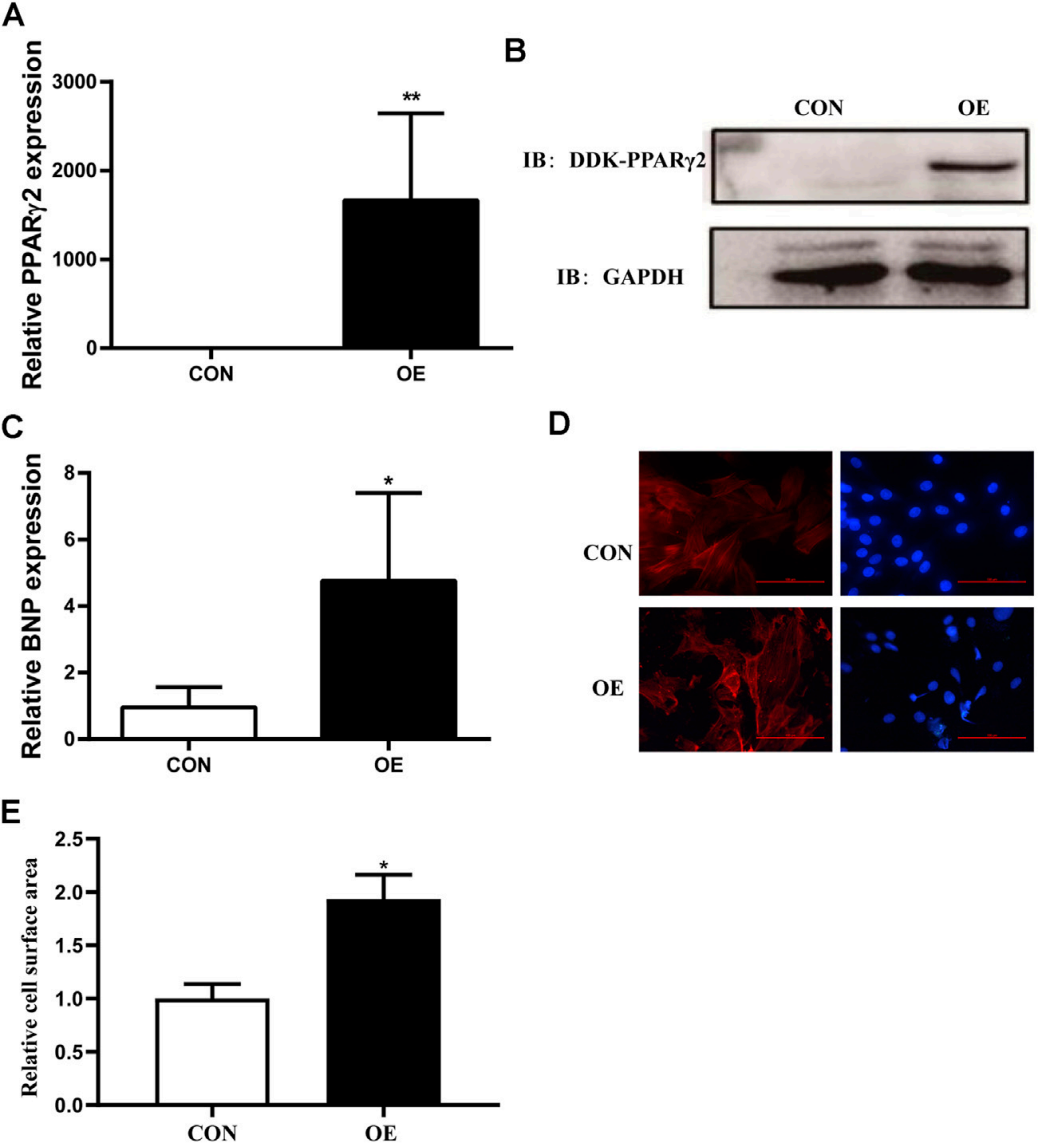
Overexpression of PPARγ2 is sufficient to lead to hypertrophy in cardiac myocytes. **(A)** Measurement of PPARγ2 mRNA levels in cardiomyocytes. **(B)** Analysis of protein levels with Western blot after overexpressing DDK-PPARγ2 in cardiomyocytes. **(C)** mRNA levels of the hypertrophic marker gene BNP after overexpressing PPARγ2 in cardiomyocytes. **(D)** representative images of α-SMA staining with CY3 (red) and nuclei with DAPI (blue) in cardiomyocytes. **(E)** quantified cell surface areas in cardiomyocytes. CON represents transfection of empty PCMV6 plasmid; OE represents transfection of the PCMV6-PPARγ2 plasmid. Means ± SEM for three independent experiments, *p < 0.05, **p < 0.01 vs. CON.

To investigate the potential role of PPARγ2 overexpression in cardiomyocytes, we transfected Myc-DDK-tagged PCMV6 and PCMV6-PPARγ2 plasmids into mouse cardiac myocytes (MCM) cells and detected the levels of PPARγ2 mRNA and protein 24 h after transfection. Results from RT-PCR and Western blotting showed that plasmids were effectively transfected (Figures 2A,B). Compared with the control (CON) group, PCMV6-PPARγ2-transfected cells showed increased BNP mRNA expression (Figure 2C). Consistent with the effects of BNP mRNA expression, the areas of cardiac myocytes were increased after labeling with α-SMA (Figures 2D,E). These results suggest that the areas and heart failure marker gene BNP were increased after overexpression of PPARγ2 in cardiac myocytes, and this should also be evidence that obesity-mediated cardiac hypertrophy and injury may be closely associated with PPARγ2 expression in the myocardium.

### Rosiglitazone and CMHX008 Displayed Different Effect on Cardiac Weight and Structure

**FIGURE 3:**
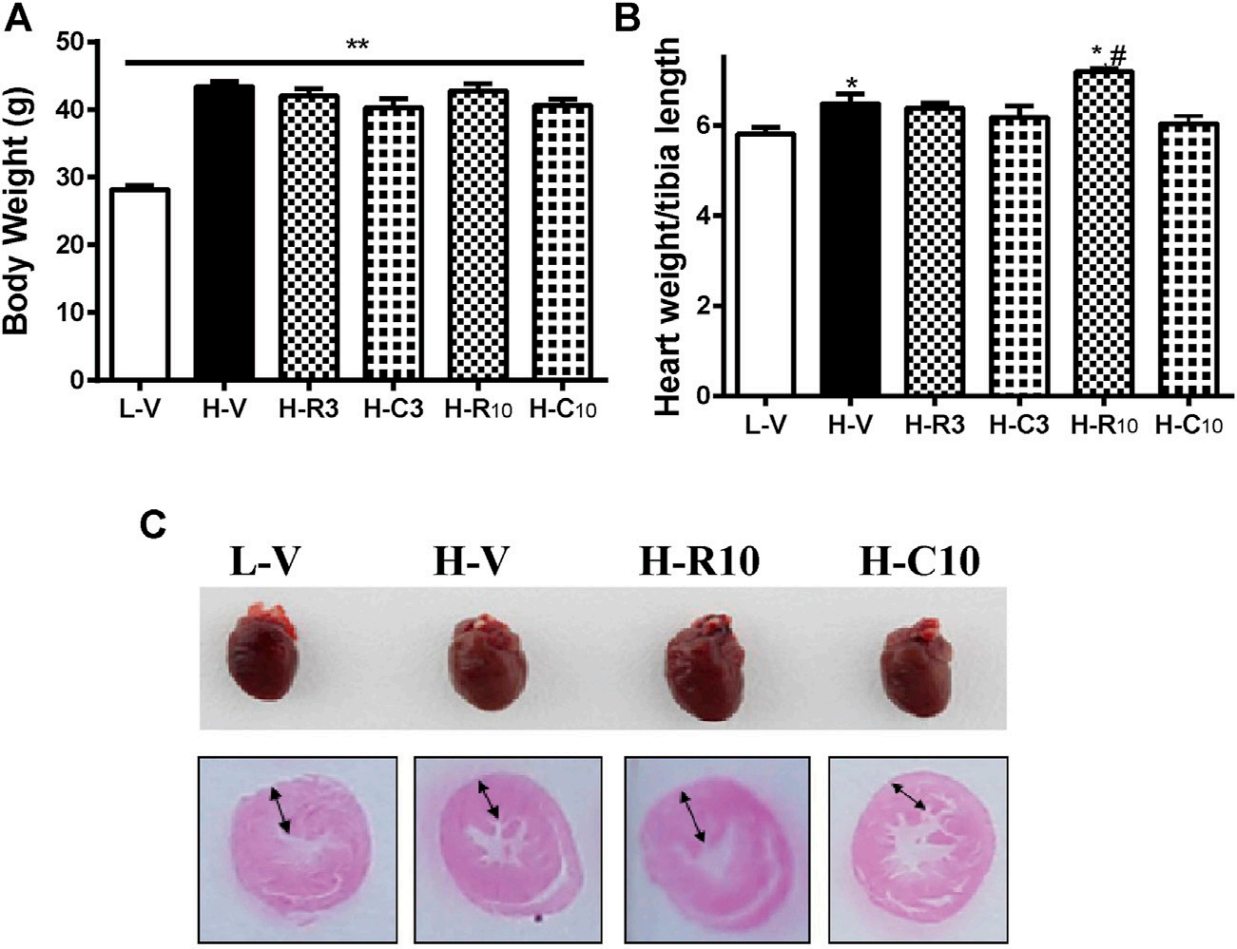
Rosiglitazone and CMHX008 displayed different changes in cardiac weight and overview morphology. **(A)** The body weight of mice treated with rosiglitazone and CMHX008 under LFD and HFD conditions; **(B)** Heart weight to body weight in LFD and HFD after treatment with rosiglitazone and CMHX008 under LFD and HFD conditions; **(C)** Macroscopic view of representative H&E–stained transverse sections from heart tissues. Means ± SEM, *n* = 6–10 mice per group, *p < 0.05, **p < 0.01 vs. L-V; ^#^ *p* < 0.05 vs. H-V.

Previous studies showed that CMHX008 displays a minor PPARγ agonist activity but with similar effects of insulin sensitization compared with rosiglitazone (Ming et al., 2014; Hou et al., 2018). After 12 weeks of treatment, there was no significant difference in body weight among rosiglitazone, CMHX008 and vehicle treated mice in HFD mice, although CMHX008 treatment showed a tendency toward lower body weight than that with rosiglitazone administration (Figure 3A). Serum cholesterol (TC) was significantly higher in the H-V group than that in the L-V group, and TC was significantly lower in the H-R10 group than that in the H-V group, but the H-C10 group showed no significant difference (Table 2). In addition, we measured the heart weight to tibia length ratio. The ratio of H-R10 mice was significantly higher than that of the other groups (Figure 3B). Cardiac hypertrophy in each group was also revealed by a macroscopic view of H&E staining in conjunction with the pictures of heart volume. Mice treated with rosiglitazone exhibited higher left ventricular wall thickness and dilated ventricles, which was not obvious with CMHX008 treatment (Figure 3C). These results suggested that rosiglitazone may induce mild cardiac hypertrophy.

**TABLE 2:** Biochemical characteristics of mice 12 weeks after intragastric administration rosiglitone or CMHX008.

	L-V	L-R_10_	L-C_10_	H-V	H-R_10_	H-C_10_
TG (mmol/L)	0.85±0.12	0.83±0.15	0.91±0.14	0.73±0.01	0.99±0.18	0.77±0.15
TC (mmol/L)	2.70±0.7	3.30±0.84	3.86±1.02	5.66±0.43 ^a^	4.62±0.7 ^a^	5.30 ± 0.18^a^
LDL (mmol/L)	0.31±0.10	0.30±0.12	0.41±0.10	0.56±0.01 ^a^	0.56±0.21 ^a^	0.48 ± 0.10^a^
BUN (mmol/L)	10.03±1.6	10.16±4.0	13.37±3.66	11.06±0.74	18.09±8.53 ^a^ ^,^ ^b^	9.11±2.79
Cr (μmol/L)	11.5±1.0	17.25±8.22	21.75±4.65	18.75±2.87	25.1±4.71 ^a^ ^,^ ^b^	12.71±5.99

Mean ± SEM, *n* = 6–8 mice per group.

a *p*< 0.05 vs. L-V.

b *p*< 0.05 vs. H-V.

### Rosiglitazone and CMHX008 Ameliorate HFD-Induced Myocardial Function but Have Different Effects on Echocardiographical Indexes

**FIGURE 4:**
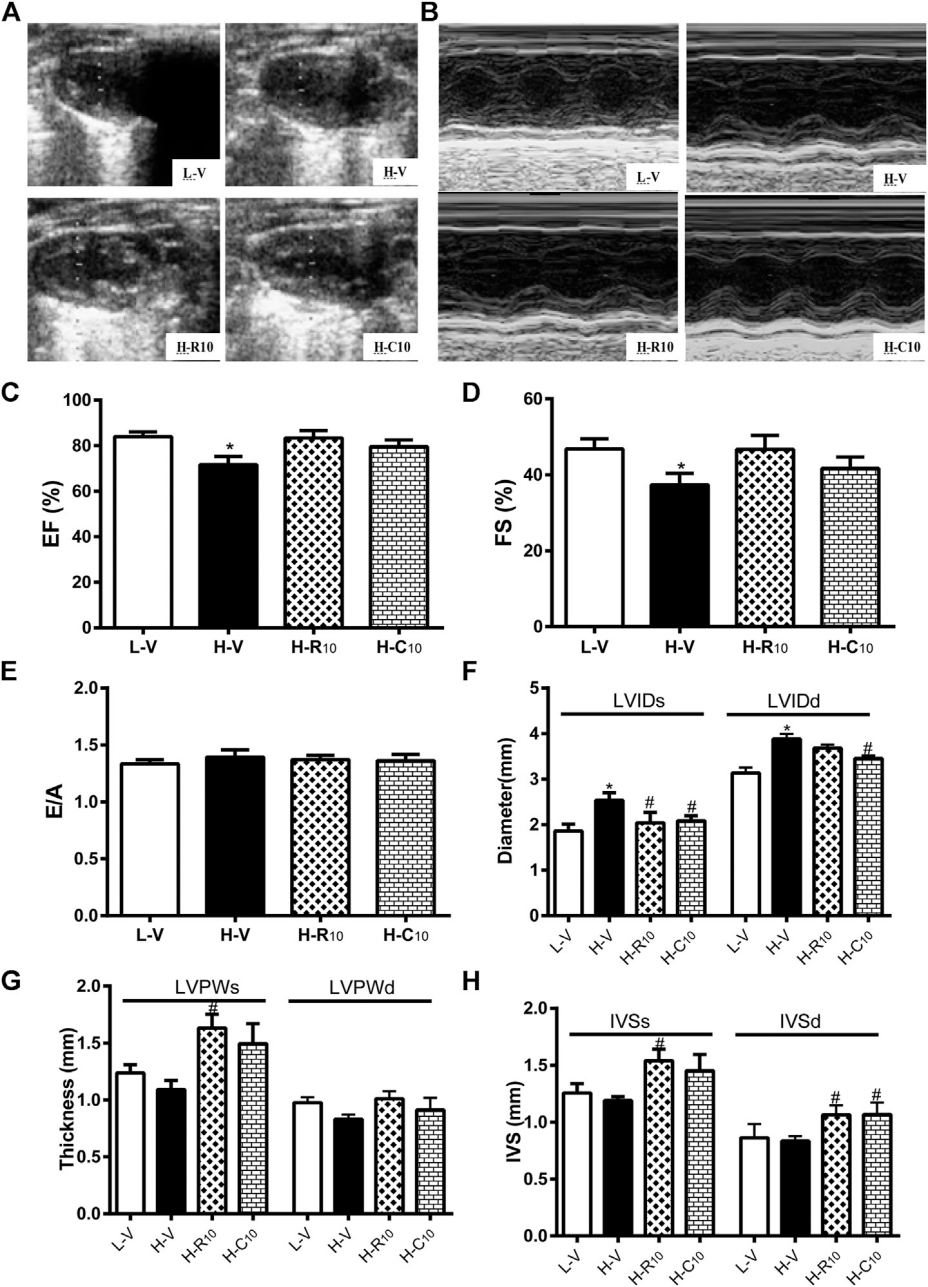
Rosiglitazone and CMHX008 ameliorate HFD-induced myocardial function but have different effects on echocardiographical indexes. **(A)** Representative 2D echocardiograph and, **(B)** M-mode image of mice treated with CMHX008, rosiglitazone or vehicle; **(C)** Quantification of ejection fraction (EF), **(D)** fractional shortening (FS), and **(E)** the ratio of E to A velocity (E/A); **(F)** Measurements of the left ventricle internal diameters in systole (LVIDs) and diastole (LVIDd), **(G)** left ventricle wall thickness in systole (LVPWs) and diastole (LVPWd), and **(H)** the interventricular septal thickness in systole (IVSs) and diastole (IVSd). Means ± SEM, *n* = 4–6 mice per group, *p < 0.05 vs. L-V, ^#^ *p* < 0.05 vs. H-V.

We then evaluated whether rosiglitazone and CMHX008 treatment could improve HFD feeding induced myocardial dysfunction by echocardiography (Figures 4A,B). Compared with vehicle-treated HFD mice (H-V), treatment with 10 mg/kg of rosiglitazone (H-R10) or CMHX008 (H-C10) significantly improved the ejection fraction (EF) and fractional shortening (FS), which were close to those of the vehicle-treated LFD control mice (L-V) (Figures 4C,D). In addition, the peak early (E) and late (A) mitral inflow velocities were measured and E/A ratio was calculated. As shown in Figure 4E, E/A showed no significant change among all groups of mice. H-R10 and H-C10 mice showed significantly decreased end-systolic diameter (LVIDs), and the left ventricle end-diastolic diameter (LVIDd) was also reduced in H-C10 group compared with H-V mice (Figure 4F). Echocardiography showed that the left ventricular end-systolic posterior wall thickness (LVPWs) was significantly thickened in the H-R10 group compared with that in the H-V mice, while the H-C10 group had a tendency of attenuating the thickening of LVPWs compared with the H-V group (Figure 4G). The left ventricular end-systolic septal thickness (IVSs) and end-diastolic septal thickness (IVSd) of the H-R10 group also showed a significant thickening, while increased IVSs was not presented in the H-C10 group (Figure 4H). Taken together, we identified that the H-R10 mice had a tendency toward cardiac hypertrophy, especially in the left ventricle posterior wall thickness and left ventricle septal thickness; however, the ejection fraction (EF) ratio and fractional shortening (FS) had not been damaged and increased reflexively. LVPW increased and EF changed slightly in the H-R10 group, which suggested that these mice were in the middle stage of diabetic cardiomyopathy and that cardiac ejection function was not damaged during this period.

### Cardiomyocyte Changes Following Treatment With CMHX008 and Rosiglitazone

**FIGURE 5:**
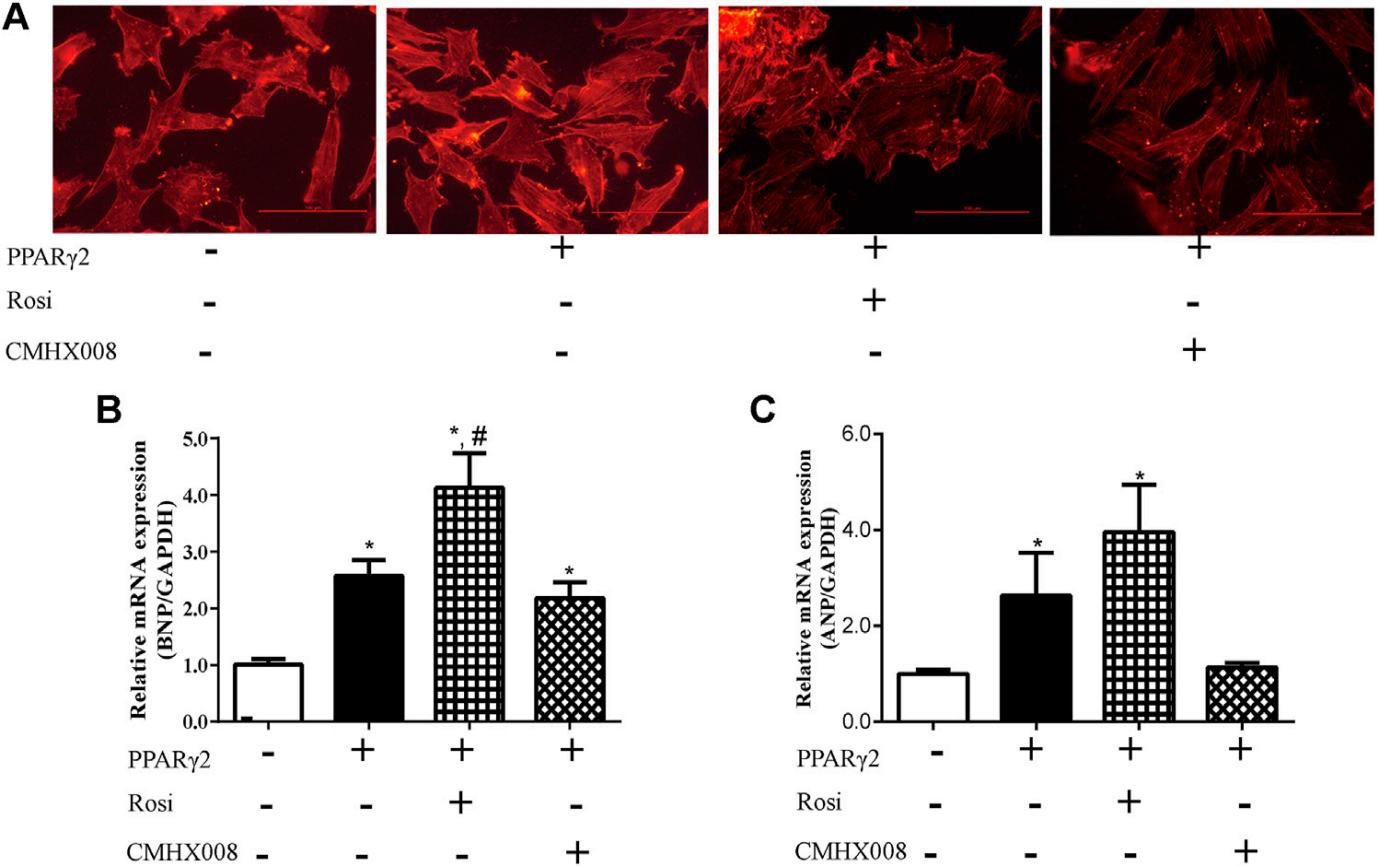
Cardiomyocyte changes following treatment with CMHX008 and rosiglitazone *in vitro*. **(A)** Representative images of α-SMA staining with CY3 (red) in cardiomyocytes; Measurement of the mRNA levels of the hypertrophic marker genes BNP **(B)** and ANP **(C)** in cardiomyocytes. Means ± SEM, from five independent experiments, *p < 0.05 vs. PPARγ2 (−); ^#^ *p* < 0.05 vs. PPARγ2 (+).

To further investigate the effect of rosiglitazone and CMHX008 on cardiomyocytes, we treated PPARγ2 overexpressed cardiomyocytes with rosiglitazone and CMHX008 and observed that rosiglitazone further aggravated hypertrophic appearance of cardiomyocytes through staining for α-SMA while CMHX008 had no obvious changes (Figure 5A). In addition, BNP and ANP, the heart failure marker genes, were highly expressed in cells treated with rosiglitazone, and the levels were higher than those in cells overexpressing PPARγ2, and cells treated with CMHX008 showed no significant difference compared with the normal group (Figures 5B,C). The results showed that rosiglitazone could aggravate the hypertrophy of cardiomyocytes induced by overexpression of PPARγ2, and CMHX008 could improve cell hypertrophy.

### The Effect of Rosiglitazone and CMHX008 on Heart Tissue of C57BL/6 Mice

**FIGURE 6:**
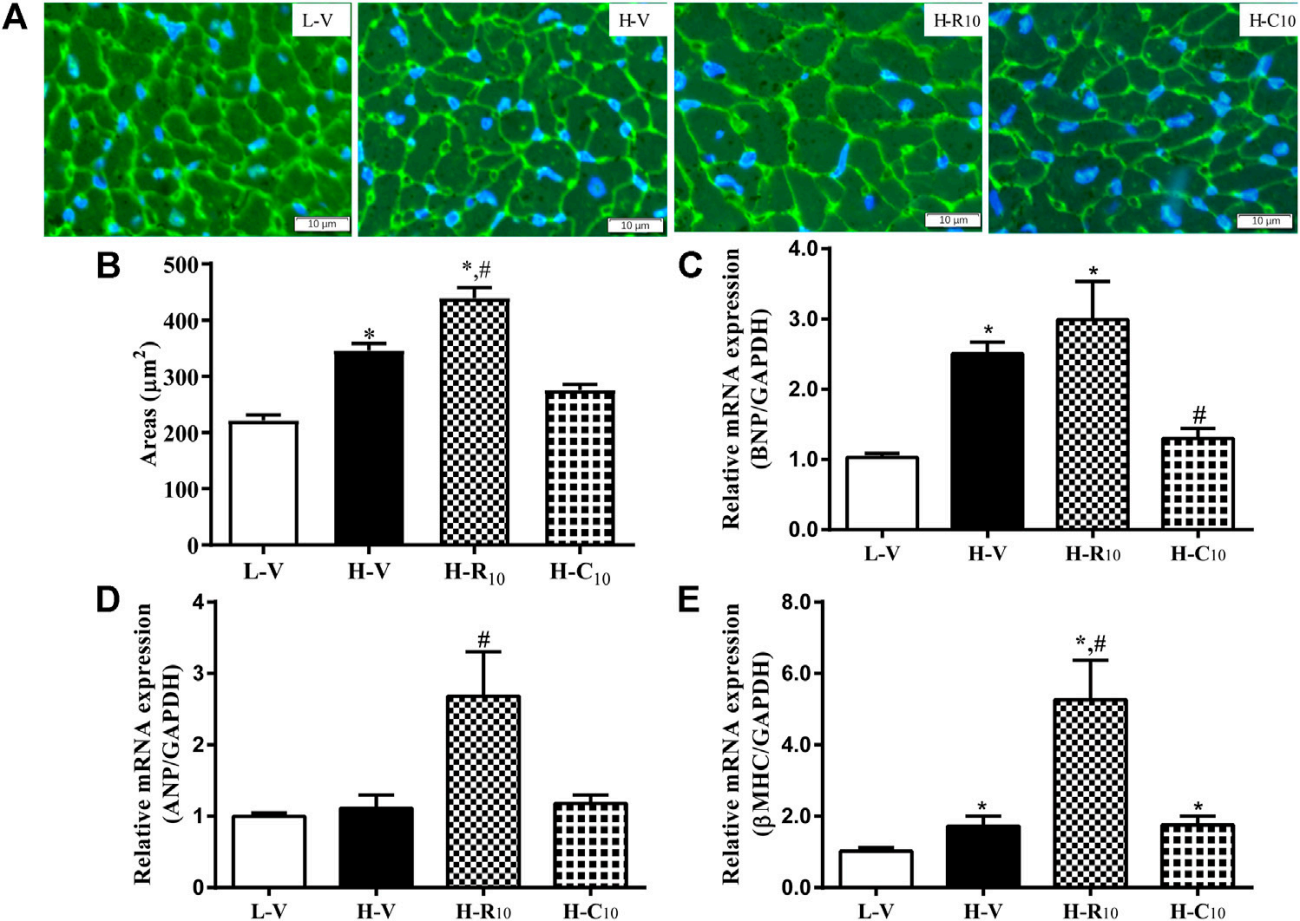
Cardiomyocyte changes in HFD mice following treatment with CMHX008 and rosiglitazone. **(A)** Representative WGA staining in the heart from HFD mice following treatment with CMHX008 and rosiglitazone; **(B)** Quantified cardiomyocyte size; Measurement of the mRNA levels of the hypertrophic marker genes BNP **(C)**, ANP **(D)**, and β-MHC **(E)** in heart tissues. Means ± SEM, *n* = 5–8 mice per group, *p < 0.05 vs. L-V, ^#^ *p* < 0.05 vs. H-V.

From WGA staining (Figures 6A,B), we observed that the cell areas were increased in the H-R10 group compared with those in the H-V group, and the cell hypertrophy in the H-C10 group was attenuated compared with the H-R10 group. In addition, the levels of heart failure-related genes, such as ANP and βMHC, were higher in the H-R10 group than the levels in the H-V group, and CMHX008 treatment reduced the expression of related gene markers compared with rosiglitazone treatment (Figures 6C–E). These results suggest that rosiglitazone may induce obvious cardiomyocyte hypertrophy and while CMHX008 treatment does not have obvious effect on cardiomyocytes.

### Patients Treated With Rosiglitazone had a High Risk of Ventricular Septal Thickness and Posterior Wall Thickness

We screened 30 patients with type 2 diabetes mellitus who had taken rosiglitazone (+ROSI group) and compared them with 90 patients as the control group with matchable age, sex and disease course but without rosiglitazone treatment (-ROSI group). Table 3 compared the data of the physical and serum biochemical characteristics. The majority of these indexes were comparable except for a modest lower ALP activity and a higher serum creatinine (Scr) level in +ROSI group. However, compared with patients without taking rosiglitazone (-ROSI), patients with rosiglitazone significantly increased, interventricular septal thickness (IVS), left ventricular posterior wall thickness (LVPW) and left atrial diameter (LAD), while left ventricular end diastolic diameter (LVDd), ejection fraction (EF) and left ventricular shortening fraction (FS) were not significantly changed, suggesting that patients with rosiglitazone had early myocardial damage and increased early volume load (Table 4).

**TABLE 3:** Physical and biochemical characteristics of the study patients.

	-ROSI	+ROSI	*p* Value
Patients (males/females)	90 (41/49)	30 (15/15)	0.673
Age (years)	60.13±10.58	63.07±12.07	0.21
Years with diabetes	7.96±5.24	7.67±5.39	0.80
Weight (kg)	61.13±11.16	63.38±7.28	0.44
BMI (kg/m^2^)	23.47±3.47	25.03±2.35	0.088
Fasting plasma glucose	9.54±3.41	8.90±2.89	0.65
HbA1c (%)	8.92±2.44	8.04±1.56	0.11
Total cholesterol (mmol/L)	4.27±1.26	4.57±0.66	0.28
TG (mmol/L)	2.05±2.94	2.05±1.10	0.75
LDL-cholesterol (mmol/L)	1.20±0.36	1.19±0.35	0.95
HDL-cholesterol (mmol/L)	2.61±0.96	2.83±0.79	0.30
Systolic blood pressure (mmHg)	133.21±19.30	135.58±19.61	0.58
Diastolic blood pressure (mmHg)	78.64±10.93	73.65±18.24	0.086
Hematocrit value (l/L)	39.04±4.80	39.03±5.96	0.99
ALP (U/L)	79.45±24.13	66.5±17.43	0.008
BUN (mmol/L)	5.88±2.20	5.95±1.58	0.23
Scr (μmol/L)	66.72±28.36	71.37±20.47	0.03

Means ± SD. −ROSI represents patients without treatment with rosiglitazone, and +ROSI represents patients treated with rosiglitazone**.**

**TABLE 4:** Echocardiographic parameters of the study patients.

	−ROSI	+ROSI	*p* Value
LV EF	63.74±5.64	64.13±3.79	0.73
LV FS	35.31±4.71	35.21±2.62	0.92
IVS	10.08±0.89	10.57±0.93	0.011 (^a^)
LVPW	9.89±0.90	10.33±0.80	0.018 (^a^)
LVDd	47.42±3.74	48±2.65	0.445
RVDd	18.91±2.02	19.13±1.91	0.60
RALD	32.68±3.06	32.97±2.44	0.63
LAD	26.84±5.06	32.97±2.44	<0.0001 (^b^)
SED	31±3.22	31.3±4.41	0.567

Means ± SD.

a *p*< 0.05.

b *p*< 0.001 vs. −ROSI.

## Discussion

The PPAR mediated transcriptional activation includes the initial activation of PPARs by ligand binding at the AF-2 region, heterodimerization with RXR, heterodimers binding to the DNA response element (PPRE), and nuclear receptor coactivator synergistic interaction with PPAR-RXR in the AF-1 region (Ma et al., 2018; Brtko and Dvorak, 2020). Several coactivators, such as CBP/p300, SRC-1, TRAP220, PGC-1α, RAP250, and cosuppressors, including NcoR and SMRT, coregulate the activity of nuclear receptors and then regulate the activity of transcription-related genes. Upon the activation of PPARγ partial agonists, the interaction of the coactivators CBP/p300 and SRC-1 with the receptor is weaker than that of PPARγ full agonists. The activation of histone acetylase by coactivators plays an important role in the regulation of different physiological responses, for example, PGC-1a plays a role in the activation of hyperglycemia, insulin resistance, and cardiomyopathy-related complexes, and transcriptional regulation of mRNA, CBP and SRC mainly regulate glucose and lipid metabolism. By TR-FRET analysis, it was confirmed that the PPARγ partial agonist F12016 had a lower recruiting ability to coactivators than the PPARγ complete agonist and reduced the side effect of bone loss (Liu et al., 2015). Other partial agonists, telmisartan, FMOC-L-Leu and PA-082, for instance, also showed a decrease in side effects, such as adipogenesis and weight gain, possibly due to differences in recruiting different coactivators (Burgermeister et al., 2006; Liu et al., 2015; Li et al., 2016; Mosure et al., 2019; Ribeiro Filho et al., 2019). These studies have implications for our study in that the different effects of CMHX008 and rosiglitazone on cardiomyocytes may be caused by the recruitment of different coactivators.

As a molecular target for thiazolidinediones, the abundance of expression and the role of PPARγ are worthy of study. The expression of PPARγ1 in streptozotocin (STZ)-induced diabetic mice was 2.1-fold higher than that in controls in heart tissues, and PPARγ1 mRNA levels in human hearts were approximately 8.1–14.5-fold higher than those in mouse hearts; these results indirectly indicated that PPARγ1 plays a functional role in the heart (Son et al., 2007). Mice with specific knockout of PPARγ exhibited modest cardiac hypertrophy, while ectopically expressing PPARγ in heart tissues also appeared to cause cardiac hypertrophy, accompanied by glucose accumulation and mitochondrial structure disorder (Krishnan et al., 2009; Luo et al., 2010; Barbieri et al., 2012). In our study, obese mice presented upregulated mRNA levels of PPARγ2 and heart failure marker genes (BNP and β-MHC) in heart tissues. Cells overexpressing PPARγ2 showed increased cell areas and increased BNP mRNA levels. Thus, we speculate that PPARγ2 is one of the key factors in diabetic cardiomyopathy.

Previously, we have clarified that CMHX008 displayed moderate PPARγ agonist activity, as it improved insulin resistance and had a lower effect on promoting preadipocyte differentiation and bone loss than rosiglitazone (Ming et al., 2014; Hou et al., 2018). To directly assess the effects of overexpression of PPARγ2, we treated HFD mice with rosiglitazone and CMHX008. As expected, by using echocardiographical examination, both rosiglitazone and CMHX008 treatment could improve myocardial dysfunction induced by HFD, with similar improvement on ejection fraction and fractional shortening. However, the *in vivo* cardiomyocyte sizes were obviously increased and transcriptional levels of hypertrophy-related genes were up-regulated in HFD mice treated with rosiglitazone while CMHX008 treatment did not demonstrate hypertrophic phenotype. The similar results were also observed in PPARγ2-overexpressed cardiomyocytes *in vitro*, showing that mouse cardiac myocytes (MCM) overexpressing PPARγ2 displayed increased levels of hypertrophy-related genes (BNP and ANP) and increased cell size after treatment with rosiglitazone but CMHX008 did not show the same effect as rosiglitazone. Rosiglitazone potentially increased the risk of cardiac hypertrophy in obese mice, and more importantly, in our clinical observation, we also found that patients with rosiglitazone had signs for early myocardial damage and increased early volume load. Our clinical observation was generally in compliance with previous findings from a systematic review and meta-analysis, showing that the relative risk of heart failure with rosiglitazone increased compared with placebo controls (Diabetes Canada Clinical Practice Guidelines Expert Committee et al., 2013; Cheng et al., 2018). It should be noted that our present study was a retrospective analysis, and thereby a population based, large-scale cohort studies with a long-term follow-up are urgently needed. In addition, the results obtained from our current study and others collectively provide a need for novel insulin sensitizers with better effectiveness and safety. CMHX008 as a novel PPARγpartial agonist showed a comparative effect on heart function with better safety compared with rosiglitazone and may potentially be developed into a new drug to benefit for T2DM with fewer cardiovascular events.

In summary, our findings show that diabetic cardiomyopathy was associated with ectopic overexpression of PPARγ2. The full agonist rosiglitazone prevents cardiac dysfunction at the expense of compensatory hypertrophy, while the partial agonist CMHX008 shared a comparable protective effect without altering the structure of cardiomyocytes.

## Data Availability

The raw data supporting the conclusions of this article will be made available by the authors, without undue reservation.

## References

[B1] Ahmadian M. Suh J. M. Hah N. Liddle C. Atkins A. R. Downes M. (2013). PPARγ Signaling and Metabolism: the Good, the Bad and the Future. Nat. Med. 19 (5), 557–566. 10.1038/nm.3159 PMC3870016 23652116

[B2] Barbieri M. Di Filippo C. Esposito A. Marfella R. Rizzo M. R. D'Amico M. (2012). Effects of PPARs Agonists on Cardiac Metabolism in Littermate and Cardiomyocyte-specific PPAR-γ-Knockout (CM-PGKO) Mice. PLoS One 7 (4), e35999. 10.1371/journal.pone.0035999 PMC3338561 22563432

[B3] Brtko J. Dvorak Z. (2020). Natural and Synthetic Retinoid X Receptor Ligands and Their Role in Selected Nuclear Receptor Action. Biochimie 179, 157–168. 10.1016/j.biochi.2020.09.027 33011201

[B4] Burgermeister E. Schnoebelen A. Flament A. Benz J. Stihle M. Gsell B. (2006). A Novel Partial Agonist of Peroxisome Proliferator-Activated Receptor-Gamma (PPARgamma) Recruits PPARgamma-Coactivator-1alpha, Prevents Triglyceride Accumulation, and Potentiates Insulin Signaling *In Vitro* . Mol. Endocrinol. 20 (4), 809–830. 10.1210/me.2005-0171 16373399

[B5] Chang C. H. McNamara L. A. Wu M. S. Muise E. S. Tan Y. Wood H. B. (2008). A Novel Selective Peroxisome Proliferator-Activator Receptor-Gamma Modulator-SPPARgammaM5 Improves Insulin Sensitivity with Diminished Adverse Cardiovascular Effects. Eur. J. Pharmacol. 584 (1), 192–201. 10.1016/j.ejphar.2007.12.036 18346728

[B6] Cheng D. Gao H. Li W. (2018). Long-term Risk of Rosiglitazone on Cardiovascular Events - a Systematic Review and Meta-Analysis. Endokrynol. Pol. 69 (4), 381–394. 10.5603/EP.a2018.0036 29952413

[B7] Chigurupati S. Dhanaraj S. A. Balakumar P. (2015). A Step Ahead of PPARγ Full Agonists to PPARγ Partial Agonists: Therapeutic Perspectives in the Management of Diabetic Insulin Resistance. Eur. J. Pharmacol. 755, 50–57. 10.1016/j.ejphar.2015.02.043

[B8] Consoli A. Formoso G. (2013). Do thiazolidinediones Still Have a Role in Treatment of Type 2 Diabetes Mellitus? Diabetes Obes. Metab. 15 (11), 967–977. 10.1111/dom.12101 23522285

[B9] Eldor R. DeFronzo R. A. Abdul-Ghani M. (2013). *In Vivo* actions of Peroxisome Proliferator-Activated Receptors: Glycemic Control, Insulin Sensitivity, and Insulin Secretion. Diabetes Care 36 (Suppl. 2), S162–S174. 10.2337/dcS13-2003 23882042 PMC3920780

[B10] Enriori P. J. Evans A. E. Sinnayah P. Jobst E. E. Tonelli-Lemos L. Billes S. K. (2007). Diet-induced Obesity Causes Severe but Reversible Leptin Resistance in Arcuate Melanocortin Neurons. Cell Metab 5 (3), 181–194. 10.1016/j.cmet.2007.02.004 17339026

[B11] Goltsman I. Khoury E. E. Winaver J. Abassi Z. (2016). Does Thiazolidinedione Therapy Exacerbate Fluid Retention in Congestive Heart Failure?. Pharmacol. Ther. 168, 75–97. 10.1016/j.pharmthera.2016.09.007 27598860

[B12] Hall J. A. Ramachandran D. Roh H. C. DiSpirito J. R. Belchior T. Zushin P. H. (2020). Obesity-Linked PPARγ S273 Phosphorylation Promotes Insulin Resistance through Growth Differentiation Factor 3. Cel Metab 32 (4), 665–e6. 10.1016/j.cmet.2020.08.016 PMC7543662 32941798

[B13] Hiatt W. R. Kaul S. Smith R. J. (2013). The Cardiovascular Safety of Diabetes Drugs-Iinsights from the Rosiglitazone Experience. N. Engl. J. Med. 369 (14), 1285–1287. 10.1056/NEJMp1309610 23992603

[B14] Home P. D. Pocock S. J. Beck-Nielsen H. Curtis P. S. Gomis R. Hanefeld M. (2009). Rosiglitazone Evaluated for Cardiovascular Outcomes in Oral Agent Combination Therapy for Type 2 Diabetes (RECORD): a Multicentre, Randomised, Open-Label Trial. Lancet 373 (9681), 2125–2135. 10.1016/S0140-6736(09)60953-3 19501900

[B15] Hou Y. Cao X. Hu X. Li X. Shi X. Wang H. (2018). CMHX008, a PPARγ Partial Agonist, Enhances Insulin Sensitivity with Minor Influences on Bone Loss. Genes Dis. 5 (3), 290–299. 10.1016/j.gendis.2018.05.004 PMC6176219 30320193

[B16] Diabetes Canada Clinical Practice Guidelines Expert Committee, Howlett J. G. MacFadyen J. C. Liu P. (2013). Treatment of Diabetes in People with Heart Failure. Can. J. Diabetes 37 (Suppl. 1), S126–S128. 10.1016/j.jcjd.2013.01.036 24070934

[B17] Janani C. Ranjitha Kumari B. D. (2015). PPAR Gamma Gene-Aa Review. Diabetes Metab. Syndr. 9 (1), 46–50. 10.1016/j.dsx.2014.09.015 25450819

[B18] Kolli V. Stechschulte L. A. Dowling A. R. Rahman S. Czernik P. J. Lecka-Czernik B. (2014). Partial Agonist, Telmisartan, Maintains PPARγ Serine 112 Phosphorylation, and Does Not Affect Osteoblast Differentiation and Bone Mass. PLoS One 9 (5), e96323. 10.1371/journal.pone.0096323 PMC4014504 24810249

[B19] Komajda M. McMurray J. J. Beck-Nielsen H. Gomis R. Hanefeld M. Pocock S. J. (2010). Heart Failure Events with Rosiglitazone in Type 2 Diabetes: Data from the RECORD Clinical Trial. Eur. Heart J. 31 (7), 824–831. 10.1093/eurheartj/ehp604 20118174 PMC2848325

[B20] Krishnan J. Suter M. Windak R. Krebs T. Felley A. Montessuit C. (2009). Activation of a HIF1alpha-PPARgamma axis Underlies the Integration of Glycolytic and Lipid Anabolic Pathways in Pathologic Cardiac Hypertrophy. Cel Metab 9 (6), 512–524. 10.1016/j.cmet.2009.05.005 19490906

[B21] Lebovitz H. E. (2019). Thiazolidinediones: the Forgotten Diabetes Medications. Curr. Diab. Rep. 19 (12), 151. 10.1007/s11892-019-1270-y 31776781 PMC6881429

[B22] Lecka-Czernik B. (2010). Bone Loss in Diabetes: Use of Antidiabetic Thiazolidinediones and Secondary Osteoporosis. Curr. Osteoporos. Rep. 8 (4), 178–184. 10.1007/s11914-010-0027-y 20809203 PMC2947013

[B23] Li H. Li M. Liu P. Wang Y. Zhang H. Li H. (2016). Telmisartan Ameliorates Nephropathy in Metabolic Syndrome by Reducing Leptin Release from Perirenal Adipose Tissue. Hypertension 68 (2), 478–490. 10.1161/HYPERTENSIONAHA.116.07008 27296996

[B24] Liu C. Feng T. Zhu N. Liu P. Han X. Chen M. (2015). Identification of a Novel Selective Agonist of PPARγ with No Promotion of Adipogenesis and Less Inhibition of Osteoblastogenesis. Sci. Rep. 5, 9530. 10.1038/srep09530 PMC4381330 25827822

[B25] Luo J. Wu S. Liu J. Li Y. Yang H. Kim T. (2010). Conditional PPARγ Knockout from Cardiomyocytes of Adult Mice Impairs Myocardial Fatty Acid Utilization and Cardiac Function. Am. J. Transl. Res. 3 (1), 61–72. PMC2981426 21139806

[B26] Ma X. Wang D. Zhao W. Xu L. (2018). Deciphering the Roles of PPARγ in Adipocytes via Dynamic Change of Transcription Complex. Front. Endocrinol. (Lausanne) 9, 473. 10.3389/fendo.2018.00473 PMC6110914 30186237

[B27] MacDonald M. R. Petrie M. C. Home P. D. Komajda M. Jones N. P. Beck-Nielsen H. (2011). Incidence and Prevalence of Unrecognized Myocardial Infarction in People with Diabetes: a Substudy of the Rosiglitazone Evaluated for Cardiac Outcomes and Regulation of Glycemia in Diabetes (RECORD) Study. Diabetes Care 34 (6), 1394–1396. 10.2337/dc10-2398 21562320 PMC3114348

[B28] Malone J. I. Hansen B. C. (2019). Does Obesity Cause Type 2 Diabetes Mellitus (T2DM)? or Is it the Opposite?. Pediatr. Diabetes 20 (1), 5–9. 10.1111/pedi.12787 30311716

[B29] Ming Y. Hu X. Song Y. Liu Z. Li J. Gao R. (2014). CMHX008, a Novel Peroxisome Proliferator-Activated Receptorγ Partial Agonist, Enhances Insulin Sensitivity *In Vitro* and *In Vivo* . PLoS One 9 (7), e102102. 10.1371/journal.pone.0102102 PMC4087031 25004107

[B30] Mosure S. A. Shang J. Eberhardt J. Brust R. Zheng J. Griffin P. R. (2019). Structural Basis of Altered Potency and Efficacy Displayed by a Major *In Vivo* Metabolite of the Antidiabetic PPARγ Drug Pioglitazone. J. Med. Chem. 62 (4), 2008–2023. 10.1021/acs.jmedchem.8b01573 PMC6898968 30676741

[B31] Nanjan M. J. Mohammed M. Prashantha Kumar B. R. Chandrasekar M. J. N. (2018). Thiazolidinediones as Antidiabetic Agents: A Critical Review. Bioorg. Chem. 77, 548–567. 10.1016/j.bioorg.2018.02.009 29475164

[B32] Ribeiro Filho H. V. Guerra J. V. Cagliari R. Batista F. A. H. Le Maire A. Oliveira P. S. L. (2019). Exploring the Mechanism of PPARγ Phosphorylation Mediated by CDK5. J. Struct. Biol. 207 (3), 317–326. 10.1016/j.jsb.2019.07.007 31319193

[B33] Rizos C. V. Kei A. Elisaf M. S. (2016). The Current Role of Thiazolidinediones in Diabetes Management. Arch. Toxicol. 90 (8), 1861–1881. 10.1007/s00204-016-1737-4 27165418

[B34] Ryan K. K. Li B. Grayson B. E. Matter E. K. Woods S. C. Seeley R. J. (2011). A Role for central Nervous System PPAR-γ in the Regulation of Energy Balance. Nat. Med. 17 (5), 623–626. 10.1038/nm.2349 PMC3089657 21532595

[B35] Saltiel A. R. (2016). New Therapeutic Approaches for the Treatment of Obesity. Sci. Transl. Med. 8 (323), 323rv2. 10.1126/scitranslmed.aad1811 26819198

[B36] Semple R. K. Chatterjee V. K. O'Rahilly S. (2006). PPAR Gamma and Human Metabolic Disease. J. Clin. Invest. 116 (3), 581–589. 10.1172/JCI28003 16511590 PMC1386124

[B37] Son N. H. Park T. S. Yamashita H. Yokoyama M. Huggins L. A. Okajima K. (2007). Cardiomyocyte Expression of PPARgamma Leads to Cardiac Dysfunction in Mice. J. Clin. Invest. 117 (10), 2791–2801. 10.1172/JCI30335 17823655 PMC1964508

[B38] Stechschulte L. A. Czernik P. J. Rotter Z. C. Tausif F. N. Corzo C. A. Marciano D. P. (2016). PPARG Post-translational Modifications Regulate Bone Formation and Bone Resorption. EBioMedicine 10, 174–184. 10.1016/j.ebiom.2016.06.040 27422345 PMC5006645

[B39] Thornton B. Basu C. (2015). Rapid and Simple Method of qPCR Primer Design. Methods Mol. Biol. 1275, 173–179. 10.1007/978-1-4939-2365-6_13 25697660

[B40] Wang L. Waltenberger B. Pferschy-Wenzig E. M. Blunder M. Liu X. Malainer C. (2014). Natural Product Agonists of Peroxisome Proliferator-Activated Receptor Gamma (PPARγ): a Review. Biochem. Pharmacol. 92 (1), 73–89. 10.1016/j.bcp.2014.07.018 PMC4212005 25083916

[B41] Yang H. I. Kim W. S. Kim D. H. Kang J. S. (2013). Histopathological Evaluation of Heart Toxicity of a Novel Selective PPAR-γ Agonists CKD-501 in Db/db Mice. Biomol. Ther. (Seoul) 21 (1), 84–88. 10.4062/biomolther.2012.101 PMC3762307 24009864

[B42] Yasmin S. Capone F. Laghezza A. Piaz F. D. Loiodice F. Vijayan V. (2017). Novel Benzylidene Thiazolidinedione Derivatives as Partial PPARγ Agonists and Their Antidiabetic Effects on Type 2 Diabetes. Sci. Rep. 7 (1), 14453. 10.1038/s41598-017-14776-0 PMC5663708 29089569

[B43] Yki-Järvinen H. (2004). Thiazolidinediones. N. Engl. J. Med. 351 (11), 1106–1118. 10.1056/NEJMra041001 15356308

